# Swyer syndrome complicated by rapidly progressive invasive ovarian dysgerminoma with bladder and rectal direct invasion: a rare case report

**DOI:** 10.3389/fonc.2026.1935665

**Published:** 2026-09-01

**Authors:** Yuan Feng, Xueqin Chen, Yaru Xu, Meng Xia, Tengda Xie, Rong Yang, Kang Du, Yiou Chen

**Affiliations:** 1 Department of Gynecology, Affiliated Qujing Hospital of Kunming Medical University/Qujing Central Hospital of Yunnan Province, Qujing, Yunnan, China; 2 Department of Neurology, Affiliated Qujing Hospital of Kunming Medical University/Qujing Central Hospital of Yunnan Province, Qujing, Yunnan, China; 3 Department of Emergency Surgery, Affiliated Qujing Hospital of Kunming Medical University/Qujing Central Hospital of Yunnan Province, Qujing, Yunnan, China

**Keywords:** 46, XY disorder of sex development, case report, ovarian dysgerminoma, primary amenorrhea, Swyer syndrome

## Abstract

A 16-year-old patient with a female social gender was admitted because of “a pelvic mass detected for one year”, accompanied by primary amenorrhea and poorly developed secondary sexual characteristics. Laboratory tests after admission showed elevated levels of follicle-stimulating hormone and luteinizing hormone, decreased estradiol level, and increased levels of β-human chorionic gonadotropin and carbohydrate antigen 125. Imaging examinations suggested multiple masses in the bilateral adnexal regions and abdominopelvic cavity. Karyotype analysis showed 46, XY. Exploratory laparotomy was performed. A huge right adnexal mass and multiple pelvic lesions were observed intraoperatively. Intraoperative frozen-section pathological examination suggested a malignant germ cell tumor, favoring dysgerminoma. Therefore, cytoreductive surgery was performed. Postoperative histopathological and immunohistochemical findings supported the diagnosis of dysgerminoma, and malignant tumor cells were detected in peritoneal lavage fluid. The final diagnosis was Swyer syndrome complicated by ovarian dysgerminoma, stage IIB. After surgery, the patient received four cycles of bleomycin, etoposide and cisplatin chemotherapy. During follow-up until April 2025, the carbohydrate antigen 125 level decreased to the normal range, and no definite recurrence was observed on imaging. This case suggests that karyotype analysis and gonadal assessment should be performed as early as possible in patients with primary amenorrhea, gonadal dysgenesis or abnormal pubertal development. Patients with Y-chromosome material are at risk of gonadal tumors. After diagnosis, multidisciplinary management involving reproductive endocrinology, gynecologic oncology, genetic counseling and psychological support is required.

## 1 Introduction

Swyer syndrome, also known as 46, XY complete gonadal dysgenesis, is a rare disorder of sex development. Patients carry a 46, XY karyotype; however, failure of embryonic gonadal differentiation toward testicular development results in predominantly female external genitalia. Müllerian structures including the uterus and fallopian tubes may be retained, while the gonads are mostly streak-like or hypoplastic. The clinical manifestations of this disease are non-specific. Most patients present with delayed puberty, primary amenorrhea or poor development of secondary sexual characteristics, which renders early diagnosis challenging (1–3). Given the presence of Y-chromosome-derived components in gonadal tissue, patients face a markedly elevated risk of germ cell tumors such as gonadoblastoma and dysgerminoma. Delayed diagnosis and intervention may lead to tumor enlargement, pelvic and abdominal dissemination, and substantially increased treatment complexity (2–4). Ovarian dysgerminoma is one of the most common malignant ovarian germ cell tumors, predominantly occurring in adolescents and young women. Although it is highly responsive to platinum-based chemotherapy, delayed diagnosis and treatment can impair long-term prognosis and quality of life (5–7). Herein, we report the diagnosis and management of one patient with Swyer syndrome complicated by ovarian dysgerminoma, followed by a literature review. This work aims to provide clinical evidence for early identification, standardized diagnosis and treatment, and long-term follow-up of patients presenting with primary amenorrhea combined with pelvic masses.

## 2 Clinical data

### 2.1 General information and medical history

The patient, socially assigned female, aged 16 years, was admitted on July 1, 2024 due to “pelvic mass detected on imaging for one year”. The patient had never experienced menarche. One year prior, she sought medical care at a local hospital, and pelvic ultrasonography revealed a cystic-solid pelvic mass measuring approximately 6.5 cm×4.2 cm, accompanied by a small uterus. She received herbal decoction and moxibustion therapy for four months afterwards, yet menarche still failed to occur.

A repeat ultrasonography at the local hospital on June 28, 2024 showed the following findings: a mixed mass slightly to the right of the pelvic cavity (14.3 cm×11.4 cm×7.5 cm) with undetermined nature, suspected teratoma requiring differentiation from other lesions; a cystic lesion within the left ovary sized 2.4 cm×2.4 cm of unknown origin; thickened left fallopian tube; small uterine volume; and pelvic effusion. Surgical intervention was recommended locally, and the patient was subsequently transferred to our department. No similar history of delayed puberty, primary amenorrhea, sexual developmental disorders or gonadal/ovarian malignant tumors was identified in the patient’s parents, siblings and three generations of direct relatives. Chromosome karyotype testing was recommended for her parents for genetic screening after discharge, and both parents showed normal 46, XX (mother) and 46, XY (father) karyotypes without pathogenic variants related to Swyer syndrome, confirming the sporadic nature of this case.

### 2.2 Physical examination

On admission, vital signs were as follows: body temperature 36°C, pulse 90 beats per minute, respiratory rate 20 breaths per minute, blood pressure 133/81 mmHg. Her height was 160 cm and body weight 59.3 kg, with a body mass index (BMI) of 23.16 kg/m^2^. Slight breast development was observed, with hypoplastic nipples and pale areolae. Pubic and axillary hair was sparse. The external genitalia presented a juvenile female morphology with intact hymenal ring; vaginal probing indicated a vaginal length of approximately 2 cm.

### 2.3 Auxiliary examinations

**Figure 1:**
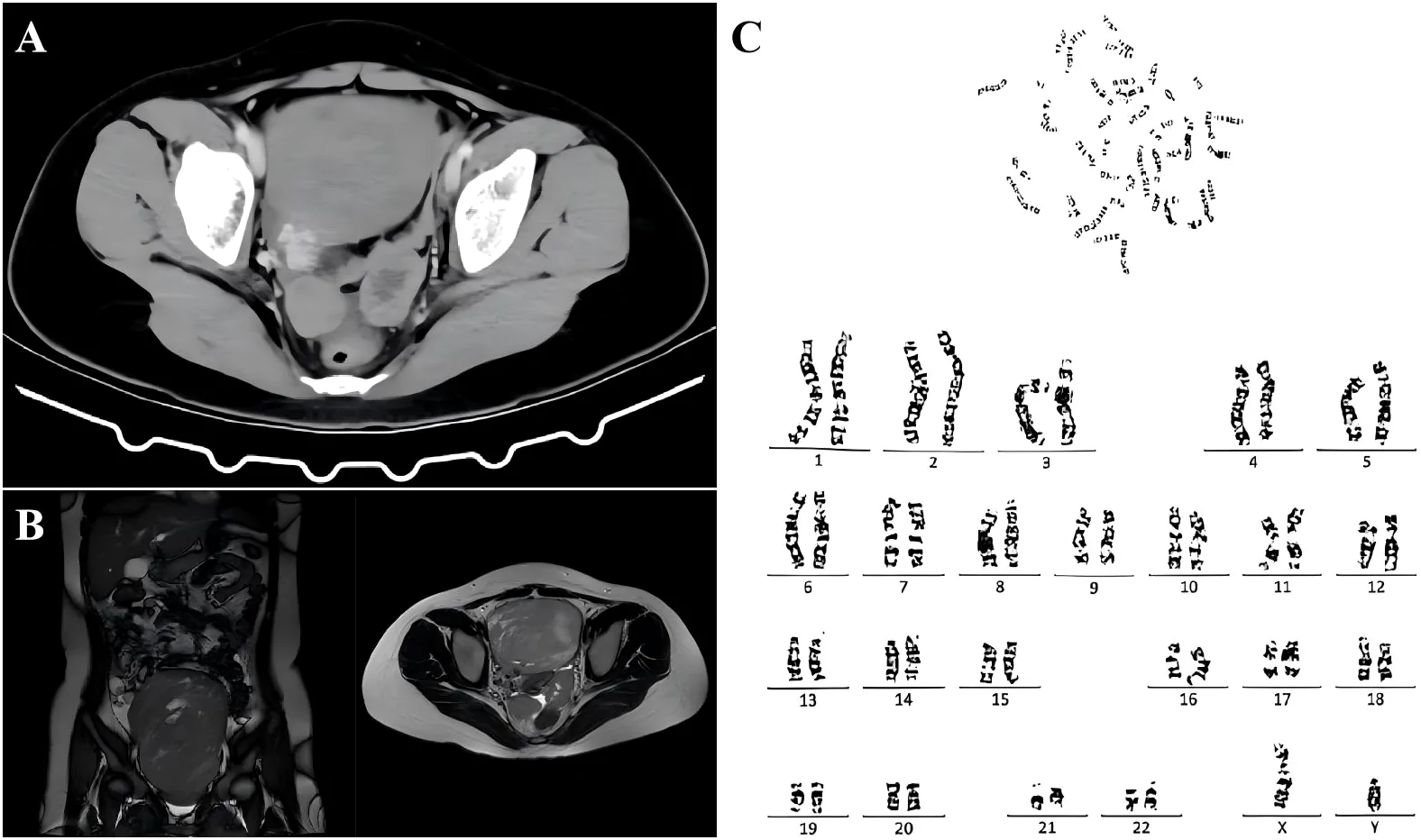
Preoperative imaging and chromosomal karyotyping results of the patient. **(A)** Preoperative contrast-enhanced chest and abdominal CT revealed bilateral ovarian masses highly suggestive of dysgerminoma, accompanied by a small right pleural effusion, mild intra-abdominal and pelvic effusions, and mild right hydronephrosis. **(B)** Coronal and axial sequences of preoperative pelvic MRI showed bilateral ovarian and abdominopelvic masses with undetermined nature, together with a small volume of pelvic fluid. **(C)** Chromosomal karyotyping confirmed a 46, XY karyotype.

Six sex hormone tests performed on July 1, 2024 yielded the following results: prolactin (PRL) 368.30 mIU/L, follicle-stimulating hormone (FSH) 80.35 mIU/mL, luteinizing hormone (LH) 38.34 mIU/mL, estradiol (E_2_) 73.35 pmol/L, testosterone (T) 0.69 nmol/L, and progesterone (P) 30.475 nmol/L. The anti-Müllerian hormone (AMH) level was 0.040 ng/mL. Tumor marker detection showed beta-human chorionic gonadotropin (β-HCG) 66.76 mIU/mL, carbohydrate antigen 125 (CA125) 545.6 U/mL, human epididymis protein 4 (HE4) 105.9 pmol/L, premenopausal ROMA index 37.55%, and postmenopausal ROMA index 79.77%. Contrast-enhanced chest and abdominal computed tomography (CT) was performed on July 2, 2024, revealing bilateral ovarian masses highly suggestive of dysgerminoma. Additional findings included a small right pleural effusion, mild intra-abdominal and pelvic effusions, and mild right hydronephrosis secondary to compression of the right ureter by the pelvic mass. No obvious abnormalities were detected in the heart, lungs, mediastinum, liver, gallbladder, pancreas, spleen, bilateral kidneys or urinary bladder (**Figure 1A**). Pelvic magnetic resonance imaging (MRI) identified bilateral ovarian and abdominopelvic masses with undetermined nature, which were suspected to be germ cell tumors, fibroblastic tumors or other lesions, accompanied by a small amount of pelvic fluid (**Figure 1B**). Chromosomal karyotyping on July 8, 2024 confirmed a 46, XY karyotype (**Figure 1C**). Preoperative diagnoses were formulated as follows: 1. Pelvic mass of unknown origin, suspected ovarian germ cell tumor; 2. 46, XY disorder of sex development, highly consistent with Swyer syndrome.

### 2.4 Surgical procedures and pathological findings

**Figure 2:**
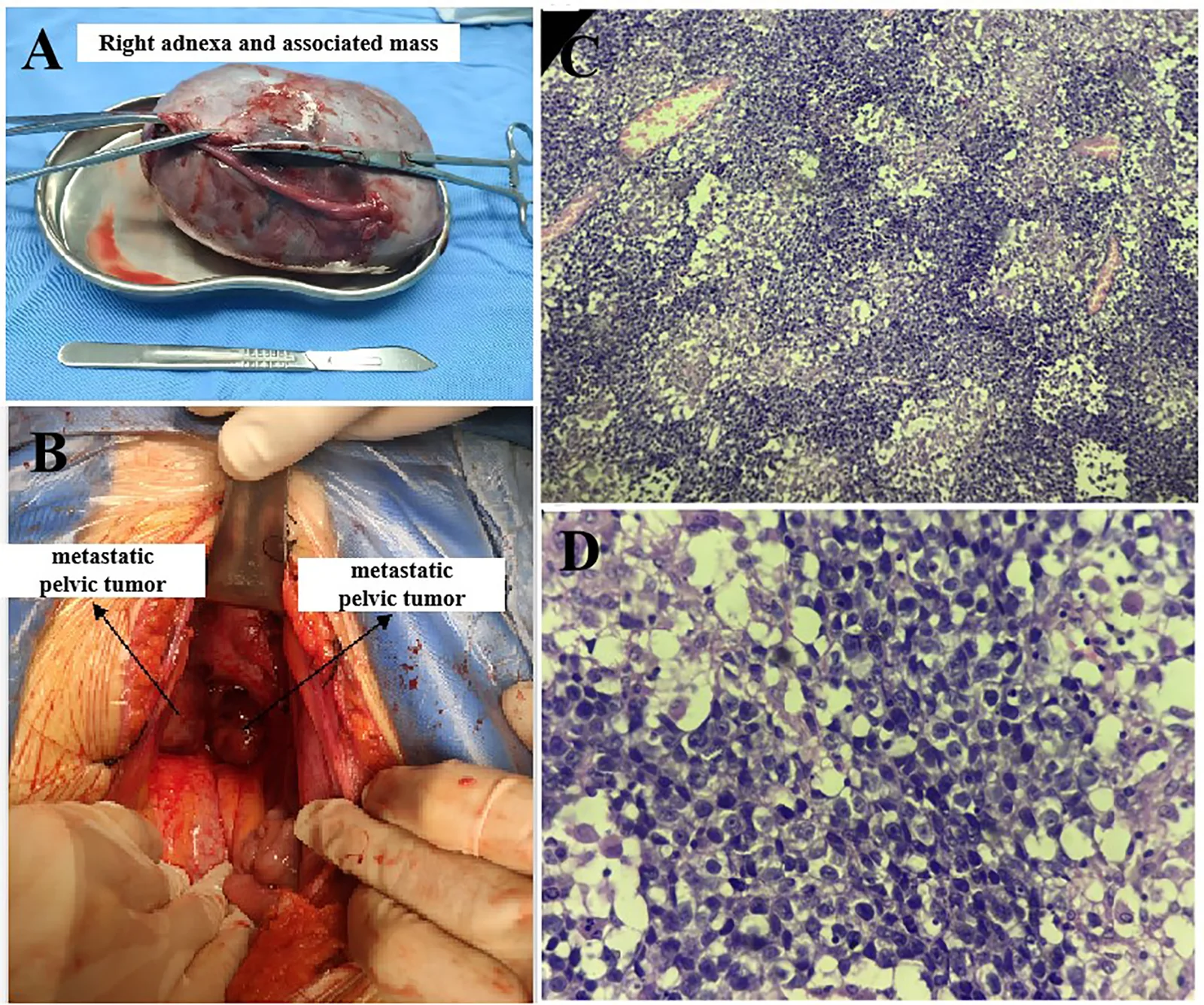
Intraoperative findings, resected specimens and pathological results of the patient. **(A)** A huge pelvic mass measuring 18 cm × 13 cm × 10 cm was observed on the resected specimen. **(B)** Intraoperative exploration identified two oval masses along bilateral uterosacral ligaments and pararectal fossae of the pelvic floor: the left lesion was 5 cm × 4 cm × 4 cm, and the right one was 4 cm × 3 cm × 3 cm. Postoperative histopathological images stained with hematoxylin-eosin (HE) **(C)** HE staining at ×100 magnification: Tumor cells arranged in sheets and nests, separated by fibrous septa with massive lymphocytic infiltration in the stroma. **(D)** HE staining at ×200 magnification: High-power view revealed tumor cells with abundant pale cytoplasm, enlarged nuclei and prominent nucleoli.

On July 23, 2024, exploratory laparotomy was performed under general anesthesia. Approximately 600 mL of bloody ascites was found intraoperatively. A huge pelvic mass measuring 18 cm×13 cm×10 cm was observed (**Figure 2A**), with relatively clear demarcation from surrounding tissues. The mass surface was rich in blood vessels, with the fallopian tube attached to it, indicating a right adnexal origin. The vascular pedicle of the mass was tortuous and dilated with abundant blood supply; the mass featured mixed texture, with solid hard areas and soft cystic regions. Further intraoperative exploration revealed a miniature rudimentary uterus sized 3 cm×2 cm×2 cm. The left fallopian tube was approximately 4 cm long with unremarkable appearance, while the left ovary exhibited abnormal morphology with only residual ovarian tissue visible. Widespread miliary lesions were identified on the vesicouterine and rectouterine peritoneal reflections, and a dark red, fragile mass measuring 4 cm×3 cm×2.5 cm was attached to the posterior wall of the urinary bladder. Two oval masses were detected along the bilateral uterosacral ligaments and pararectal fossae of the pelvic floor: the left lesion was 5 cm×4 cm×4 cm, and the right one was 4 cm×3 cm×3 cm (**Figure 2B**). During dissection, these masses showed indistinct boundaries with the rectum and bilateral pelvic sidewalls. No tumor lesions were palpable in the liver, gallbladder, pancreas, spleen, intestines, greater omentum or appendix, and no enlarged pelvic or para-aortic lymph nodes were identified.

Intraoperative frozen-section pathology indicated a malignant germ cell tumor of the right pelvic mass, most consistent with dysgerminoma. Light microscopy (×100/×200 magnification): Tumor cells proliferated in large sheets and island nests, separated by fibrous connective tissue septa with massive diffuse lymphocyte infiltration in stroma. Tumor cells had abundant pale eosinophilic cytoplasm, enlarged round/oval nuclei, thick nuclear membrane and obvious prominent nucleoli; mitotic figures could be occasionally observed, without glandular, yolk sac or embryonic carcinoma differentiation components (**Figures 2C, D**). Immunohistochemistry: Tumor cells diffusely positive for CD117, SALL4, PLAP and D2-40; completely negative for CD30, GPC-3, AFP, α-Inhibin and Calretinin. Proliferation index Ki-67 was about 60%. The above immunohistochemical profile confirmed the pathological diagnosis of pure ovarian dysgerminoma. Malignant tumor cells were detected in the peritoneal lavage fluid. After intraoperative communication with the patient’s family, cytoreductive surgery was carried out, including resection of the giant pelvic mass, total abdominal hysterectomy, bilateral salpingo-oophorectomy, high ligation of ovarian arteries and veins, resection of rectal lesions, and excision of bladder lesions.

### 2.5 Diagnosis, treatment and follow-up

Postoperative diagnoses were confirmed as follows: 1. Malignant tumor of the right ovary, dysgerminoma stage IIB (T2bN0M0); 2. 46, XY disorder of sex development consistent with Swyer syndrome, with female social gender; 3. Direct tumor invasion of the rectum; 4. Direct tumor invasion of the urinary bladder; 5. Malignant ascites. Four cycles of chemotherapy with the bleomycin, etoposide and cisplatin (BEP) regimen were administered postoperatively. The dosage regimen was bleomycin 15 U/m^2^, etoposide 167 mg/m^2^, and cisplatin 33 mg/m^2^. Tumor marker levels declined progressively throughout treatment (**Table 1**). A follow-up contrast-enhanced CT scan performed at our hospital on April 17, 2025 revealed postoperative changes after pelvic mass resection, with absence of the uterus and bilateral adnexa. Pelvic exudative effusion was almost completely absorbed compared with prior imaging; slight thickening of the pelvic floor fascia remained unchanged, and the lower rectal segment showed significant dilatation. Plain and contrast-enhanced CT scans of the liver, gallbladder, pancreas, spleen, bilateral kidneys and urinary bladder showed no remarkable abnormalities. Concurrent reexamination showed CA125 decreased to 11.85 U/mL.

**Table 1:** Changes in tumor markers before surgery and during chemotherapy.

Timeline	β-HCG (mIU/mL)	CA125 (U/mL)	HE4 (pmol/L)	Pre-menopausal ROMA index (%)	Post-menopausal ROMA index (%)
2024-07-01 (Preoperation)	66.76	545.60	105.90	37.55	79.77
2024-08-19 (1st cycle of chemotherapy)	1.88	64.36	51.80	8.75	28.16
2024-09-10 (2nd cycle of chemotherapy)	0.947	22.18	46.10	6.36	13.74
2024-10-04 (3rd cycle of chemotherapy)	0.589	14.51	44.30	5.68	10.07
2024-10-30 (4th cycle of chemotherapy)	0.497	15.48	56.40	9.70	11.31
2025-04-17 (Follow-up examination)	–	11.85	–	–	–

β-HCG, beta-human chorionic gonadotropin; CA125, carbohydrate antigen 125; HE4, human epididymis protein 4.

The symbol “-” indicates that relevant data were not available in the original records.

## 3 Discussion

Swyer syndrome, also termed 46, XY complete gonadal dysgenesis, represents a rare subtype of disorders of sex development. First reported by Swyer in 1955, this condition is characterized by a 46, XY karyotype, yet embryonic gonads fail to differentiate into testes. Clinically, patients typically present with female external genitalia and preserved Müllerian structures including the uterus and fallopian tubes, whereas the gonads appear streak-like or hypoplastic (1–3). Owing to the absent normal endocrine function of dysplastic gonads, most patients seek medical care during adolescence for primary amenorrhea, underdeveloped secondary sexual characteristics, or delayed puberty. Michala et al. (4) described 29 patients with Swyer syndrome, among whom 26 presented predominantly with delayed puberty. This finding indicates that early identification of this disease largely relies on heightened vigilance toward abnormal pubertal development and primary amenorrhea. The present patient, aged 16 years, had never experienced menarche. Physical examination revealed inadequate development of breasts, pubic hair and axillary hair. Auxiliary tests demonstrated elevated follicle-stimulating hormone and luteinizing hormone alongside low estradiol levels. Imaging detected a small uterus and an adnexal mass, and chromosomal karyotyping yielded a 46, XY result, consistent with the diagnostic criteria of Swyer syndrome.

Clinical manifestations of Swyer syndrome are non-specific, and diagnosis is easily delayed if relying solely on external genital phenotypes. For phenotypically female patients presenting with primary amenorrhea, poor development of secondary sexual characteristics, a hypoplastic uterus or adnexal masses, comprehensive examinations should be performed as early as possible, including sex hormone profiling, anti-Müllerian hormone detection, pelvic ultrasonography or magnetic resonance imaging, tumor marker assays, and chromosomal karyotyping (2–4). A pelvic mass was identified in the present patient one year prior to admission, yet systematic endocrine, genetic and gynecologic oncologic evaluations were not conducted in a timely manner. By admission, the tumor had markedly enlarged, accompanied by ascites and multiple pelvic lesions. This case suggests that when primary amenorrhea is complicated by a pelvic mass, clinicians should not merely manage the case as functional amenorrhea or a benign adnexal mass; 46, XY disorders of sex development and gonad-derived tumors must be included in the differential diagnosis.

Patients with Swyer syndrome carry a markedly elevated risk of gonadal neoplasms, primarily attributed to the retention of Y-chromosome-related material within hypoplastic gonads. Previous studies have demonstrated that individuals with disorders of sex development harboring Y-chromosome material are susceptible to gonadoblastoma, dysgerminoma and other germ cell tumors (5, 6). In the cohort of 29 Swyer syndrome patients reported by Michala et al. (4), gonadal pathological specimens were available for 22 individuals, among whom 7 were diagnosed with dysgerminoma and 3 with gonadoblastoma; the youngest patient with dysgerminoma was only 10 years old. The research by Matsumoto et al. (5) also indicated that patients carrying Y-chromosome material with bilateral streak gonads face a high risk of gonadal malignancy, and prophylactic gonadectomy should be prioritized upon diagnosis. Lu et al. (6) analyzed phenotypically female children and adolescents with Y-chromosome-positive disorders of sex development, and identified a high incidence of gonadal tumors among patients with 46, XY complete gonadal dysgenesis. The authors recommended early prophylactic gonadectomy immediately after confirmed diagnosis.

This case stands out for its rapid disease progression and extensive tumor dissemination, which sharply differs from previous literature reports. Most previously documented cases of Swyer syndrome complicated with neoplasms detected early-stage tumors incidentally during prophylactic gonadectomy, or presented with tumors confined solely to the gonads. In contrast, the pelvic mass in this patient expanded drastically from 6.5 cm to 18 cm within merely one year, accompanied by rare direct invasive metastasis to the bladder and rectum as well as malignant ascites. Such rapid progression from an occult lesion to stage IIB advanced invasive malignant tumor within a short time frame is extremely rare in existing publications. Furthermore, the mass was misdiagnosed as teratoma at initial detection, and non-surgical management was attempted, which not only delayed the optimal intervention window but also reflected insufficient clinical vigilance toward malignant tumors complicating this syndrome. This case strongly suggests that any pelvic lesion identified in patients with Swyer syndrome should be managed as a high-risk malignant lesion regardless of its initial size, and a multidisciplinary collaborative diagnosis and treatment protocol should be initiated immediately to prevent catastrophic tumor dissemination resulting from watchful waiting or misdiagnosis.

Ovarian dysgerminoma is one of the major pathological subtypes of malignant ovarian germ cell tumors, predominantly occurring in adolescents and young women. Its clinical manifestations include abdominopelvic mass, abdominal pain, abdominal distension, menstrual irregularities, or elevated serum tumor markers (7–10). Elevated serum β-HCG can be detected in a subset of patients with dysgerminoma, while increased CA125 is associated with large tumor volume, peritoneal irritation, ascites formation, or pelvic dissemination. Several cases of Swyer syndrome complicated with dysgerminoma have been documented in previous literature. Han et al. (7) reported a patient with 46, XY complete gonadal dysgenesis who presented with a pelvic mass and was pathologically diagnosed with dysgerminoma after surgery. Milewicz et al. (8) described a patient who failed to receive regular follow-up after initial consultation and developed abdominal distension years later; postoperative pathology confirmed concurrent dysgerminoma and gonadoblastoma. Collectively, these previously reported cases together with the present case indicate that patients with Swyer syndrome who do not undergo timely resection of hypoplastic gonads are at risk of developing germ cell tumors that may progress to large lesions. Definitive diagnosis of dysgerminoma relies on pathological morphology and immunohistochemistry. Typical histological features include tumor cells arranged in sheets, nests or trabeculae with abundant pale cytoplasm, accompanied by fibrous septa and lymphocytic infiltration in the stroma. Immunohistochemically, dysgerminoma consistently expresses markers including PLAP, CD117, OCT3/4, SALL4 and D2-40, whereas negative expression of CD30, GPC-3, AFP and sex cord-stromal tumor markers helps distinguish it from embryonal carcinoma, yolk sac tumor and sex cord-stromal neoplasms (9). Postoperative pathology and immunohistochemistry of the present case revealed positive staining for CD117, SALL4, PLAP and D2-40, and negative staining for CD30, GPC-3, α-Inhibin and Calretinin, which confirmed the diagnosis of dysgerminoma. For patients with clinically suspected malignant germ cell tumors, preoperative testing of LDH, AFP, β-HCG and CA125 is recommended to facilitate tumor subtyping, therapeutic response evaluation and postoperative surveillance (6, 9, 10).

Upon diagnosis of Swyer syndrome with confirmed Y-chromosome material, patients and their guardians should be fully informed of the risk of gonadal neoplasms, and individualized management strategies should be formulated based on social gender, age, malignancy risk, as well as the wishes of patients and guardians. For patients without malignant transformation, resection of hypoplastic gonads is generally recommended to reduce the risk of gonadoblastoma and dysgerminoma (2–6). When malignant germ cell tumors have developed, surgical and adjuvant therapeutic regimens should be tailored according to tumor stage, lesion extension, patient age, social gender and fertility demands. Malignant ovarian germ cell tumors are generally sensitive to platinum-based regimens. The BEP combination chemotherapy serves as one of the standard adjuvant postoperative regimens (6, 10–12). Williams et al. (11) conducted research on postoperative adjuvant BEP chemotherapy for ovarian germ cell tumors and demonstrated satisfactory disease control in most patients. Dimopoulos et al. (12) also validated the efficacy and safety of the BEP regimen in this tumor type. Intraoperative exploration of our patient revealed a massive right adnexal mass, multiple pelvic lesions and malignant ascites. Postoperative diagnosis was Swyer syndrome complicated with stage IIB ovarian dysgerminoma. After four cycles of BEP chemotherapy, the levels of β-human chorionic gonadotropin, carbohydrate antigen 125 and ROMA index decreased markedly, and no definite recurrence was detected on follow-up CT scans, indicating favorable short-term therapeutic efficacy.

Long-term management of Swyer syndrome extends far beyond anti-tumor therapy; clinicians should also attach importance to sex hormone replacement, bone mineral density preservation, reproductive counseling and psychological support. Michala et al. (4) identified osteopenia in a subset of Swyer syndrome patients, and rational hormone replacement therapy helps maintain bone mineral density and may improve uterine volume and morphology. For patients with an intact uterus and well-controlled tumor, fertility can be achieved in the future via assisted reproductive technology using donor oocytes or embryos (4, 13, 14). Therefore, for patients with localized tumors, uninvolved uterus and fertility preservation demands from themselves and guardians, a multidisciplinary team should comprehensively evaluate the feasibility of uterine preservation prior to surgery. Given the extensive pelvic lesions in this patient, total hysterectomy and bilateral salpingo-oophorectomy were performed. Subsequent management priorities include surveillance for tumor recurrence, estrogen replacement therapy, bone metabolism regulation, long-term toxicity assessment of chemotherapy, and psychological intervention.

This case possesses distinctive clinical features: the pelvic tumor rapidly grew from 6.5 cm to 18 cm in 12 months, accompanied by uncommon direct invasion into the bladder and rectum with malignant ascites (stage IIB), which was far more aggressive than localized gonadal dysgerminoma previously reported in Swyer syndrome patients. Besides, early misdiagnosis as benign teratoma and conservative herbal treatment delayed definitive surgery. Meanwhile, this study has several limitations: the follow-up only lasted 6 months post BEP chemotherapy without long-term surveillance data; targeted genetic sequencing for gonadal dysgenesis (SRY, NR5A1, etc.) was unavailable due to financial constraints, failing to elucidate its molecular pathogenesis; moreover, being a single rare advanced case, multi-center cohort studies are needed to confirm the incidence and risk factors of rapidly progressive dysgerminoma among Swyer syndrome patients. To facilitate subsequent management, we formulated a standardized long-term follow-up protocol consisting of quarterly tumor marker testing, semiannual abdominopelvic contrast-enhanced CT, annual bone mineral density and sex hormone examinations, plus lifelong genetic and psychological follow-up.

In conclusion, although Swyer syndrome is a rare disease, it constitutes a critical differential diagnosis for patients presenting with primary amenorrhea, underdeveloped secondary sexual characteristics, a hypoplastic uterus, or adnexal masses. Clinicians should deepen their understanding of 46, XY disorders of sex development, and perform chromosomal karyotyping and gonadal assessment at an early stage. For patients carrying Y-chromosome material, clinicians should proactively evaluate the risk of gonadal neoplasms and administer prophylactic or therapeutic surgery in a timely manner. Long-term surveillance is mandatory following treatment for dysgerminoma. Follow-up protocols should include pelvic and abdominal imaging examinations, serial detection of tumor markers including β-human chorionic gonadotropin, lactate dehydrogenase, alpha-fetoprotein and carbohydrate antigen 125, as well as evaluation of long-term chemotherapy-related toxicities. Given that this disease involves sexual development, social gender identity, reproductive potential and malignant tumor management, multidisciplinary collaboration covering reproductive endocrinology, gynecologic oncology, genetic counseling, assisted reproduction and psychological medicine should be strengthened throughout diagnosis and treatment.

## Data Availability

The original contributions presented in the study are included in the article/supplementary material. Further inquiries can be directed to the corresponding authors.
